# Application of omental flap in total vaginectomy for post-radiotherapy recurrent cervical cancer: a case report

**DOI:** 10.3389/fsurg.2026.1779661

**Published:** 2026-04-10

**Authors:** Haixia Shu, Min Wang, Lixia Zhang, Xiaoling Jiang, Wei Yang, Xiaoli Mo, Chunmei Li, Lianli He

**Affiliations:** 1 Department of Gynecology and Obstetrics, The Third Affiliated Hospital of Zunyi Medical University (The First People's Hospital of Zunyi), Zunyi, Guizhou, China; 2 Department of Imaging, The Third Affiliated Hospital of Zunyi Medical University (The First People's Hospital of Zunyi), Zunyi, Guizhou, China

**Keywords:** case report, cervical cancer, greater omentum, pedicled flap, vaginal cancer

## Abstract

Cervical squamous cell carcinoma (CSCC) is one of the most common gynecological malignancies, and radiotherapy is a key treatment modality. This paper reports a case of a 51-year-old female patient with cervical squamous cell carcinoma who developed a recurrence in the vaginal wall following radiotherapy. The patient was diagnosed with stage IIIb cervical squamous cell carcinoma four years ago and underwent radical radiotherapy at another hospital. Treatment consisted of pelvic external beam radiotherapy (50 Gy in 25 fractions) combined with two-dimensional brachytherapy (6 Gy in 4 fractions), yielding a total EQD2 of 82 Gy. The treatment proceeded without complications, and no significant radiation-induced damage was observed; however, the patient did not attend regular follow-up appointments after radiotherapy. During this follow-up, squamous cell carcinoma (keratinising type) of the vaginal wall was identified. Following a comprehensive evaluation that ruled out surgical contraindications, the patient underwent laparoscopic total hysterectomy, bilateral salpingo-oophorectomy, and total vaginectomy combined with vaginal packing using a pedicled omental flap. Given the presence of radiotherapy-induced vaginal fibrosis, total vaginectomy carries a heightened risk of poor wound healing, as well as an increased risk of bladder and bowel injuries, which would be challenging to manage in a subsequent repair. To promote healing of the vaginal stump and prevent the occurrence of vesicovaginal and rectovaginal fistulas, the vaginal stump was packed with an omental flap during the procedure. The pedicled omentum flap has a rich blood supply and lymphatic drainage; when packed into the vaginal stump during surgery, it effectively promotes local tissue healing, forms a biological barrier to isolate the bladder from the rectum, and significantly reduces the incidence of fistula formation. A full abdominal and pelvic CT scan performed three months post-operatively revealed no significant abnormalities in the vaginal wall, providing preliminary confirmation of the procedure's safety and efficacy. Given that the clinical management of such patients remains challenging, this case offers a new technical reference for the diagnosis and treatment of similar cases, emphasising the importance of individualised, multidisciplinary, comprehensive treatment, and holds potential clinical value for improving the prognosis of these patients.

## 1 Introduction

The management of cervical squamous cell carcinoma encompasses a range of modalities, including surgery, radiotherapy, and chemotherapy. In accordance with the latest domestic guidelines, the treatment strategy for newly diagnosed patients strictly adheres to the FIGO staging system and is further tailored based on age, fertility requirements, and pathological type to formulate an individualized approach. The core principle is “surgery as the mainstay, complemented by radiotherapy and chemotherapy, and integrated with immunotherapy and targeted therapy when indicated.” However, despite standardized treatment, patients still face a certain risk of recurrence. The patterns of recurrence may vary but typically include local recurrence (LR) and distant metastases (DM). The management of recurrent disease requires a personalized approach based on the site of recurrence, which may determine the prognosis (1). Previous studies have shown that the prognosis of patients following initial treatment is associated with clinical and pathological characteristics, the pattern of recurrence, and the type of treatment administered for the recurrence (2–4).

Reportedly, the recurrence rate after primary treatment ranges from 25% to 61% (5), and among patients who previously received concurrent chemoradiotherapy, the recurrence rate is 20%–40% (6). A multicenter retrospective study from Italy, involving 327 women with recurrent cervical cancer, indicated that the most common sites of recurrence were local (vaginal) and regional. Following radical surgery, the most frequent site of recurrence was central pelvic.

Here, we report a case of vaginal wall recurrence in a patient with cervical squamous cell carcinoma who had previously undergone radiotherapy.

## 2 Case report

**Figure 1:**
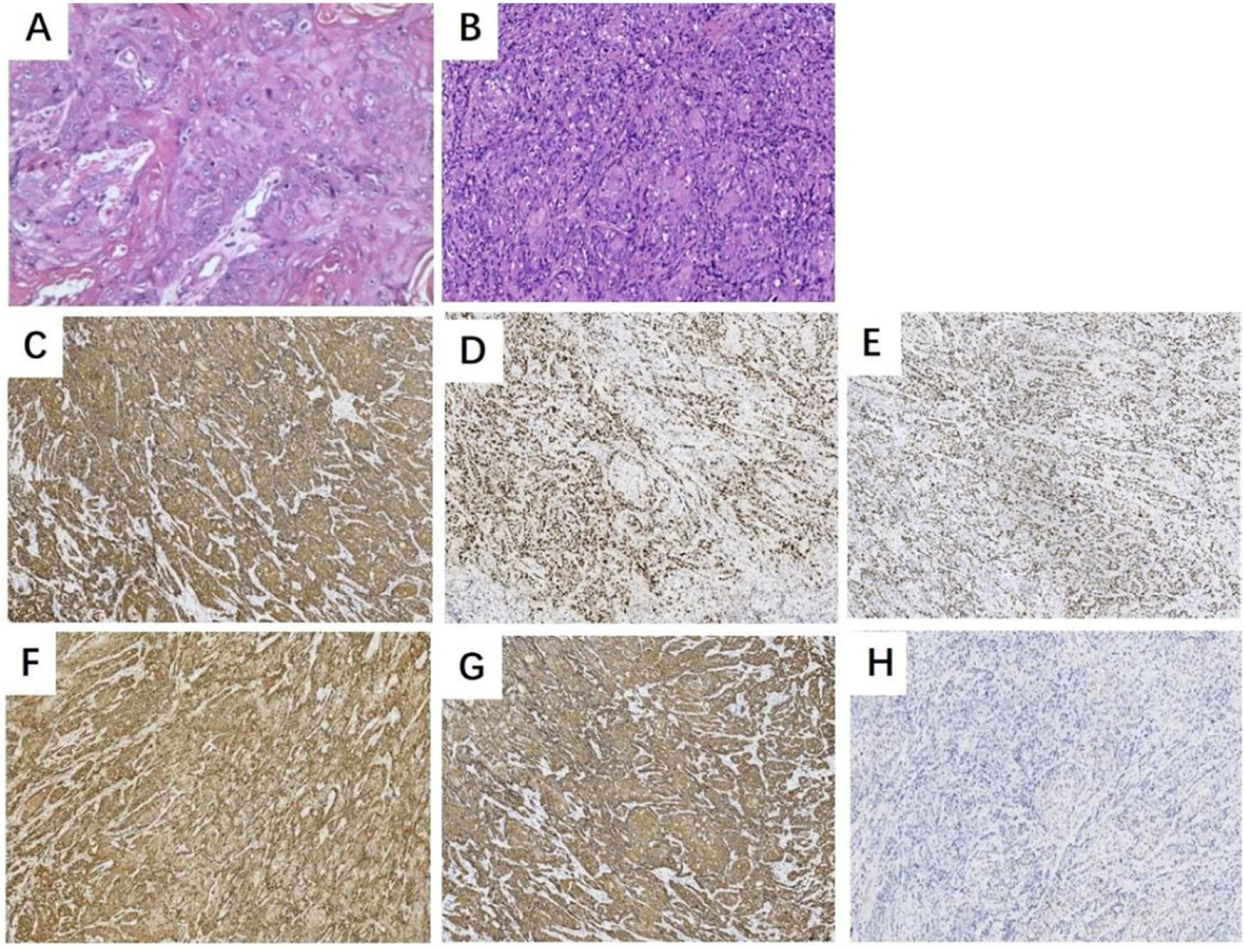
Pathological findings: **(A)** HE staining of squamous cell carcinoma of the cervix: tumor cells were distributed in nests, with diffuse growth and pathological nuclear division; **(B)** HE staining of highly-moderately differentiated squamous cell carcinoma of the vaginal wall: tumor cells were dense, losing original tissue structure, with pathological nuclear division and large cellular anisotropy. Immunohistochemistry: tumor cells in highly-moderately differentiated squamous cell carcinoma (vaginal wall) showed diffuse positivity for **(C)** CK5/6, **(D)** CK14, **(E)** p63, **(F)** p16, and **(G)** Ki-67, and **(H)** p53 was not expressed.

**Figure 2:**
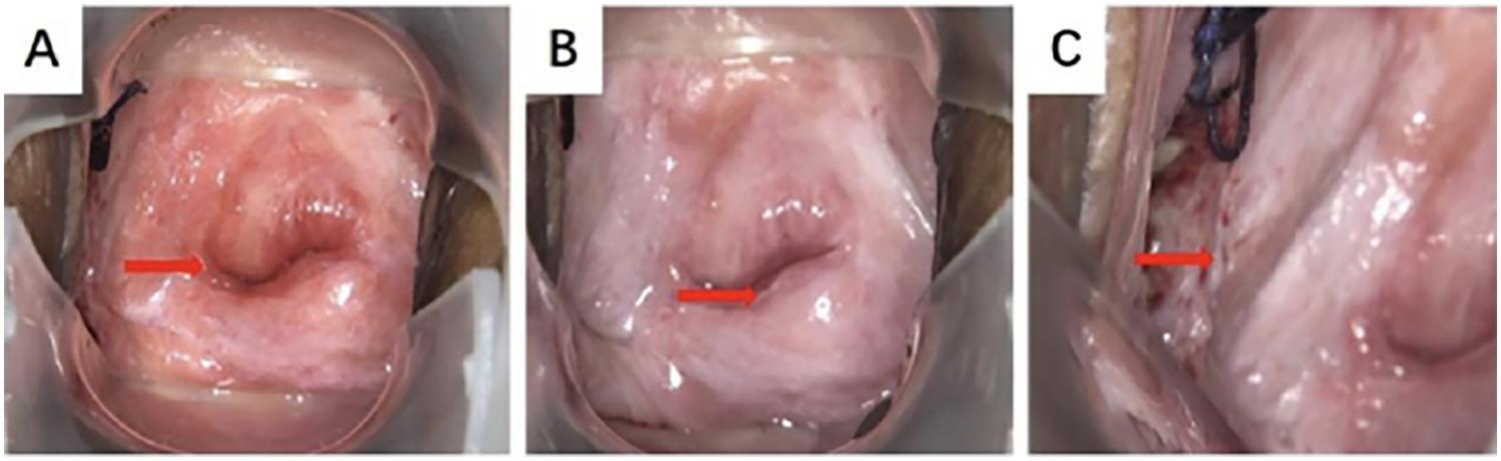
Colposcopy: **(A)** hyperplasia-like tissue of about 0.5 cm was seen at point 9 of the lower cervix by colposcopy. **(B)** Vinegar-white test: thin vinegar-white epithelium. **(C)** The right vaginal wall was lacerated, about 1 cm long, and a small amount of white tissue was seen on the trauma.

**Figure 3:**
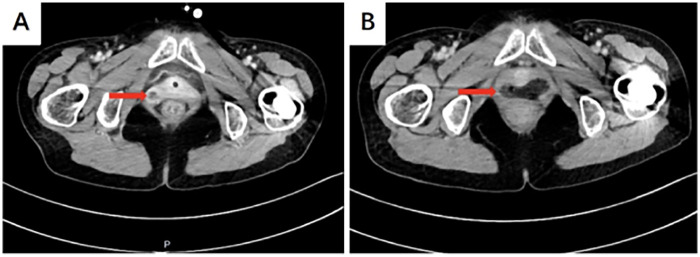
Whole abdomen + pelvis CT: **(A)** a mass is seen at the junction of the middle and lower 1/3 of the right side wall of the vagina, with a maximum diameter of about 22 mm (red arrow); **(B)** no obvious abnormality is seen in the vaginal wall: (red arrow).

A 51-year-old female was admitted to the hospital with “recurrent vaginal bleeding for half a year”. The patient is a 41-year-old postmenopausal woman, no vaginal bleeding and fluid after menopause, G2P2 (full-term delivery of a son and a daughter), denying the family history of heredity and tumors. 4 years ago, cervical cancer was diagnosed in a foreign hospital, and the pathology results suggested: Cervical squamous cell carcinoma (Figure 1A), and then in a hospital in Chongqing, she received radiotherapy 28 times. Examination: blood staining of the vulva, about 50 g blood clot in the posterior vaginal dome, about 2 × 1 cm ulcerated surface on the right wall of the vagina, accompanied by active bleeding, cervical atrophy, no bleeding, and ulceration. The uterus was anterior with no pressure, the cervix had no lifting or swinging pain, the posterior fornix had no tenderness, the bilateral adnexa had no detectable masses and no pressure, and there was no thickening or shortening of the sacral ligaments bilaterally in the triple diagnosis. Laboratory examination: HIV (+), squamous epithelial cell carcinoma antigen 1.80 ng/mL (higher than normal). Auxiliary examination: colposcopy showed hyperplasia-like tissue of about 0.5 cm at point 9 of the cervix (Figure 2A); vinegar-white test: thin vinegar-white epithelium (Figure 2B); a laceration of the right vaginal wall of about 1 cm in length, with a small amount of white tissue visible on the surface of the wound (Figure 2C). Pelvic CT suggested that a mass was seen at the junction of the middle and lower 1/3 of the right vaginal wall, with a maximum diameter of about 22 mm (Figure 3A), which was considered to be a recurrence in the vaginal wall after radiotherapy for cervical cancer. To further utilize PET/CT for disease staging. Colposcopic biopsy suggested: (right vaginal wall tissue) squamous cell carcinoma (keratinized type); (cervical points 3 and 6) chronic cervicitis with some areas of squamous epithelial high-grade intraepithelial lesion (HSIL/CINIII grade) and involvement of glands; (cervical point 9) free squamous epithelial high-grade intraepithelial lesion (HSIL/CINIII grade).

**Figure 4:**
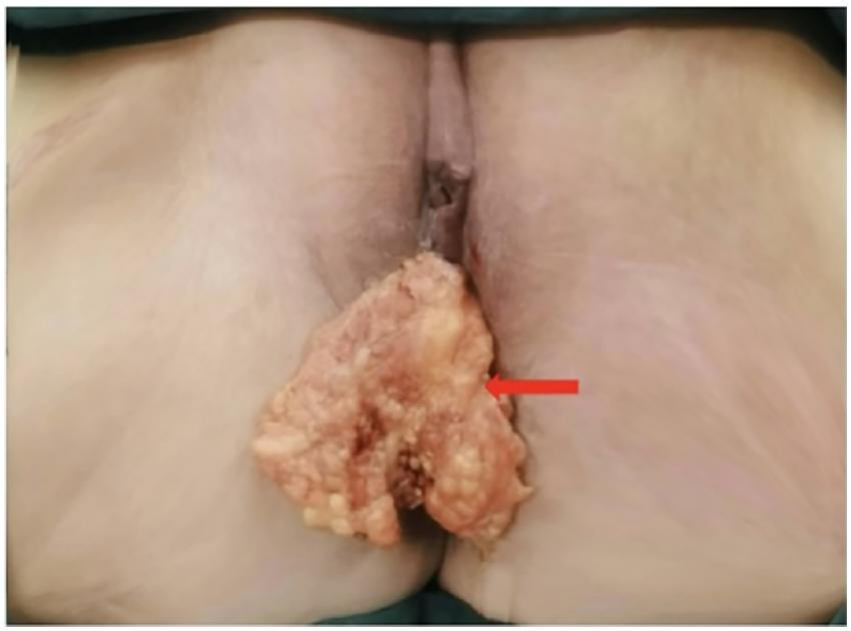
Intraoperative tamponade of an intact greater omental flap into the vagina (red arrow).

Based on the patient's medical history, physical examination, and auxiliary findings, the following diagnoses were considered: 1. Recurrent cervical malignancy following radiotherapy; 2. HIV positive. After ruling out surgical contraindications, the patient underwent surgical treatment. Intraoperative examination confirmed that the lesion was confined to the vaginal wall; a thorough exploration of the uterus, bilateral adnexal regions, and the pelvic and abdominal cavities revealed no significant abnormalities. The planned radical surgery was then performed: total hysterectomy, bilateral salpingo-oophorectomy, and total vaginectomy. A pedicled omentum flap was used to cover the vaginal stump during the same procedure: first, the omentum at the level of the transverse colon was clamped to determine the resection margin; subsequently, a portion of the omentum was precisely incised using an ultrasonic scalpel in the avascular area lateral to the greater gastric artery arch, and the omentum flap was carefully dissected, taking care to preserve the integrity of the gastric omental vascular arch. The omental flap was then pulled downwards along the natural space of the right paracolic gutter and carefully placed over the vaginal stump to prevent enterocutaneous fistula, promote healing of the vaginal stump, and prevent infection. Intraoperative blood loss was approximately 200 mL, and the total duration of the procedure was 6 h. The omentum located at the vaginal opening usually detaches spontaneously after surgery (Figure 4). Postoperative pathological findings confirmed that the vaginal wall mass was a highly moderately differentiated squamous cell carcinoma (Figure 1B), and the tumor invaded more than 2/3 of the entire vaginal wall, with no definite vascular or neural invasion, and there was no tumor involvement of the severed ends of the vaginal wall, (left and right) parietal margins, (left and right) pelvic margins, uterine corpus, (bilateral) fallopian tubes, and (bilateral) ovarian tissues. Immunohistochemical tests showed: CK5/6 (+) (Figure 1C), CK14 (+) (Figure 1D), p63 (+) (Figure 1E), p16 (+) (Figure 1F), Ki-67 (+, about 60%) (Figure 1G) and p53 (-) (Figure 1H).

Postoperative pathological examination revealed a moderately differentiated squamous cell carcinoma of the vaginal wall, with tumor infiltration involving more than two-thirds of the full thickness of the vaginal wall. No definite vascular or perineural invasion was observed. The resection margins, including the proximal and distal vaginal ends, bilateral parietal margins, and bilateral parametrial margins, were free of tumor involvement, as were the uterine corpus, bilateral fallopian tubes, and ovarian tissues. These findings were consistent with a vaginal recurrence of cervical cancer. Preoperative imaging showed a vaginal mass located at the junction of the middle and lower thirds of the right vaginal wall, with a maximum diameter of approximately 22 mm, and no evidence of distant metastasis. Pathological assessment confirmed the absence of vascular or perineural invasion, and the surgical resection achieved radical margins. Based on these findings, no adjuvant therapy was deemed necessary, and regular follow-up was recommended.

## 3 Discussion

### 3.1 Differentiation of vaginal recurrence from vaginal primary in cervical cancer

Recurrent cervical cancer (CC) refers to tumor regrowth after curative treatment and most commonly occurs in patients with advanced-stage disease. It remains clinically challenging to manage and is associated with a poor prognosis, representing a leading cause of death among cervical cancer patients (7, 8). The most frequent sites of recurrence can be categorized as follows (9): local (vaginal vault or paravaginal region), regional (pelvic lymph nodes), and distant (extrapelvic sites, including para-aortic lymph nodes, distant organs, bone, muscle, and peritoneum). Vaginal recurrence of CC typically presents with symptoms such as irregular vaginal bleeding and pelvic pain. Vaginal malignancies may be classified as primary or secondary. Primary vaginal cancer originates in the vagina without concurrent cervical or vulvar involvement and is rare, accounting for only 1%–2% of all gynecologic malignancies. Secondary vaginal cancer results from the spread of malignant cells from other sites, such as cervical cancer, to the vagina. Thus, primary vaginal cancer and recurrent cervical cancer involving the vagina represent two distinct clinical entities. According to the International Federation of Gynecology and Obstetrics (FIGO), the diagnosis of primary vaginal carcinoma requires the absence of a tumor in the cervix or vulva and is further contingent upon the following temporal criteria: (1) at least 2 years after treatment for cervical carcinoma *in situ*; (2) at least 5 years after surgery for invasive cervical cancer; or (3) at least 10 years after radiotherapy for cervical cancer.

Research indicates that HIV infection is associated with an increased incidence of cervical cancer and accelerated disease progression (10); The patient was diagnosed as HIV-positive four years ago; given this, it is likely that the condition will continue to affect the patient's immune function, tumour prognosis and wound healing. However, since being diagnosed, the patient has consistently followed medical advice and undergone standard antiretroviral therapy (ART). A comprehensive assessment of the patient's immune status was carried out prior to surgery. The results indicated that the viral load was well controlled, suggesting good immune function and that the patient was fit to undergo surgery. Given that HIV-positive patients have approximately twice the risk of surgical site infection compared to HIV-negative individuals (11), enhanced perioperative infection prevention measures were implemented. These included strict aseptic technique during surgery and prophylactic antibiotic administration postoperatively to reduce the risk of incisional infection. The patient has recovered well following the operation and has not developed any infectious complications. The patient in this case presented primarily with vaginal bleeding, had a history of HIV infection, and had undergone radiotherapy for cervical squamous cell carcinoma four years prior. Furthermore, postoperative pathology revealed that the mass in the vaginal wall was a moderately to well-differentiated squamous cell carcinoma; the patient is currently considered to have a central-type recurrence of cervical cancer (involving the vaginal wall).

### 3.2 Treatment of vaginal recurrence of cervical cancer

Treatment of vaginal recurrence of cervical cancer: For patients with recurrent cervical cancer who have previously undergone radiotherapy and chemotherapy, the standard treatment usually involves a modified radical hysterectomy or a pelvic exenteration. A conservative hysterectomy is usually recommended when the surgical margins are likely to be clear and the tumor is primarily confined to the uterus; however, a pelvic exenteration is usually required when the recurrence involves adjacent organs such as the bowel or bladder. Previous studies have found that vaginectomy is considered a treatment option for isolated vaginal recurrence in cervical cancer (CC) (9). In this case, the patient was found to have a recurrence of cervical cancer in the vaginal wall following radiotherapy. To ensure the patient's quality of life after surgery and reduce the risk of recurrence, following a multidisciplinary team (MDT) discussion, we opted for a surgical approach comprising laparoscopic total hysterectomy, bilateral salpingo-oophorectomy, total vaginectomy, and vaginal reconstruction using a pedicled omentum flap. As the patient had undergone a total vaginal resection, a pedicled omentum flap was used to reconstruct the vagina in order to augment the vaginal tissue and protect the bladder and bowel. Given the presence of radiotherapy-induced tissue fibrosis and the patient's compromised healing capacity, the risk of poor stump healing would be significantly increased without the use of an omental flap.

### 3.3 The advantages of the greater omentum with its pedicle

The greater omentum consists of four layers of peritoneum; it resembles an apron and covers the anterior surfaces of the jejunum, ileum, and transverse colon. It originates from the greater curvature of the stomach, extends downwards to the anterior surface of the transverse colon, then curves backwards and upwards to join the mesentery of the transverse colon. A potential space exists between the anterior and posterior layers of the greater omentum, known as the omental bursa. The decision to use a pedicled omental flap rather than a VRAM, a thin fascial flap, or a simple peritoneal flap for pelvic reconstruction is primarily based on the following clinical considerations: Firstly, the greater omentum flap offers the advantage of being minimally invasive, thereby avoiding the donor site damage and functional loss associated with muscle-skin flaps (12). For patients who require preservation of abdominal wall function, the greater omentum flap does not require the sacrifice of the rectus abdominis muscle, thereby reducing the risk of abdominal wall hernias and abdominal wall protrusions. For cancer patients who have undergone curative surgery and radiotherapy or chemotherapy, preserving the integrity of the abdominal wall is crucial to their quality of life after surgery. Secondly, the omentum is rich in a network of lymphatic vessels and possesses powerful immune defence functions and the ability to absorb interstitial fluid (13). It is capable of absorbing serum, lymph and blood from the pelvic cavity, whilst simultaneously promoting the migration of white blood cells to combat bacteria. In contaminated or infected environments, this characteristic offers an advantage over muscle flaps. Furthermore, the greater omentum flap possesses angiogenic properties (14, 15), enabling it to provide nutritional support and blood supply to the recipient tissue. In the ischemic environment following radiotherapy, the rich blood supply of the greater omentum can promote capillary ingrowth and improve tissue healing. The greater omentum also offers advantages in terms of functional reconstruction (16). It effectively isolates the small intestine from the pelvic wound, reducing the risk of intestinal obstruction and fistula formation, and facilitates ureteral reconstruction and the repair of complex fistulas (such as vesicovaginal and rectovaginal fistulas) (8); However, omentum flaps have limitations, such as significant inter-individual variation in tissue volume and an inability to provide structural support; furthermore, in an oncological context, they may be associated with a higher risk of recurrence ^(^17, 18). Clinical decisions must therefore take into account the patient's body type, previous surgical history, history of radiotherapy and oncological risk, and reconstruction strategies must be selected on an individual basis.

### 3.4 Principles of treatment for recurrent cervical cancer

Treatment of recurrent cervical cancer includes surgery, radiotherapy, and chemotherapy (19). Relevant studies have shown that systemic therapy with or without radiation is the basis of treatment for patients with recurrent or metastatic disease (20). The patient's admission pathology results (tissue from the right vaginal wall) indicated cervical squamous cell carcinoma (keratinizing type), representing a central recurrence within the radiotherapy field. As the patient had previously undergone radiotherapy, and taking into account her wishes and her HIV-positive status—which would compromise her tolerance to further radiotherapy—surgical treatment is considered the preferable option. Chemotherapies for cervical cancer include single-agent chemotherapy and combination chemotherapy (21), and the standard chemotherapy regimen for recurrent cervical cancer is platinum-based combination chemotherapy. Platinum drugs are antitumor drugs that, upon binding to the DNA of tumor cells, inhibit their DNA replication, thereby reducing tumor volume (22). Although there are many chemotherapy regimens for recurrent and metastatic cervical cancer, the current first-line chemotherapy regimen is cisplatin/carboplatin + paclitaxel + bevacizumab. The 2018 NCCN guidelines recommend pembrolizumab as a second-line treatment for recurrent and metastatic cervical cancer (23, 24), but in recurrent cervical cancer, chemotherapy is usually not used alone and is usually more effective in combination with other agents (25). Meanwhile, according to the NCCN Clinical Practice Guidelines for Cervical Cancer (2023 Edition), (1) for local recurrence: ① if the patient has not received radiotherapy before, the first choice is synchronized radiotherapy and chemotherapy (external irradiation radiotherapy + brachytherapy + cisplatin chemotherapy); (2) if the patient has received radiotherapy before, surgical treatment (e.g., pelvic contouring) or systemic treatment (chemotherapy ± targeted therapy) can be considered. ③ For locally recurrent diseases, systemic therapy is primarily employed when local treatment modalities are not feasible. (2) For distant metastasis or extensive recurrence: ① Systemic treatment is the mainstay, and chemotherapy combined with bevacizumab (anti-angiogenic drug) is recommended; ② for PD-L1-positive patients, immunotherapy (e.g., pembrolizumab) can be considered (26).

### 3.4 Follow-up

Three-month post-operative outpatient follow-up: The abdominal incision has healed well, with no signs of redness, swelling, or oozing; the vaginal stump has healed well, with no abnormal bleeding. A CT scan of the entire abdomen and pelvis showed no obvious masses or signs of recurrence in the vaginal wall; the serum squamous cell carcinoma antigen (SCC-Ag) level was 0.80 ng/mL, which is within the normal reference range. The patient reports that their quality of life since the operation has been good, with no significant discomfort. Overall assessment: The response to treatment has been good, and the condition is stable; it is recommended that regular follow-up appointments continue.

Follow-up was assessed by gynecological examination, vaginal exfoliative cytology (cervical exfoliative cytology for those with preserved cervix), serum tumor markers (e.g., blood squamous epithelial cell carcinoma antigen), and imaging. Follow-up visits should be scheduled every 3 to 6 months for the first 2 years after treatment, every 6 to 12 months for the 3rd to 5th year, and annually thereafter. Follow-up intervals should be shortened for high-risk patients, while intervals for low-risk patients may be appropriately extended (2024 NCCN Oncology Guidelines for Clinical Practice on Cervical Cancer) (27).

## 4 Conclusion

In addition, clinicians should further deepen their knowledge of vaginal recurrence of cervical cancer to reduce the rate of misdiagnosis and underdiagnosis. Accurately determining whether a patient's vaginal wall mass is primary or neoplastic is extremely important for the precise management of this disease. Developing highly individualized treatment plans for different patients to achieve optimal outcomes is a key goal in clinical practice. In order to effectively treat and manage such diseases, close multidisciplinary collaboration is essential. Currently, there is a lack of clear guidelines and standardized surgical procedures for vaginal wall recurrence of cervical cancer. In this case, we used a comprehensive surgical protocol of laparoscopic total hysterectomy combined with double adnexectomy, total vaginectomy, and vaginal tamponade with a large omental flap. Therefore, with this case, we hope to provide useful reference and guidance for the management of similar cases in the clinic.

## Data Availability

The original contributions presented in the study are included in the article/supplementary material, further inquiries can be directed to the corresponding author.
